# Highly Sensitive Readout Interface for Real-Time Differential Precision Measurements with Impedance Biosensors

**DOI:** 10.3390/bios13010077

**Published:** 2023-01-02

**Authors:** Sara Neshani, Kasra Momeni, Degang J. Chen, Nathan M. Neihart

**Affiliations:** 1 Electrical and Computer Engineering Department, University of Alabama, Tuscaloosa, AL 35401, USA; 2 Mechanical Engineering Department, University of Alabama, Tuscaloosa, AL 35401, USA; 3 Electrical Engineering Department, Iowa State University, Ames, IA 50010, USA

**Keywords:** real-time, impedance biosensor, low-cost, precision, sensitive, readout, interface

## Abstract

Field deployment is critical to developing numerous sensitive impedance transducers. Precise, cost-effective, and real-time readout units are being sought to interface these sensitive impedance transducers for various clinical or environmental applications. This paper presents a general readout method with a detailed design procedure for interfacing impedance transducers that generate small fractional changes in the impedance characteristics after detection. The emphasis of the design is obtaining a target response resolution considering the accuracy in real-time. An entire readout unit with amplification/filtering and real-time data acquisition and processing using a single microcontroller is proposed. Most important design parameters, such as the signal-to-noise ratio (SNR), common-mode-to-differential conversion, digitization configuration/speed, and the data processing method are discussed here. The studied process can be used as a general guideline to design custom readout units to interface with various developed transducers in the laboratory and verify the performance for field deployment and commercialization. A single frequency readout unit with a target 8-bit resolution to interface differentially placed transducers (e.g., bridge configuration) is designed and implemented. A single MCU is programmed for real-time data acquisition and sine fitting. The 8-bit resolution is achieved even at low SNR levels of roughly 7 dB by setting the component values and fitting algorithm parameters with the given methods.

## 1. Introduction

Recently, real-time sensing and detection are revolutionizing many services such as healthcare, home automation, transportation, etc. Moreover, there is a push to develop more field-deployable biosensors for medical diagnostics and environmental monitoring applications. Motivated by this, there has been a surge in the development of new transducers targeting a wide range of biosensing applications for detecting proteins, ions, temperature, etc. 

Label-free impedance biosensors are famous prototypes for point-of-care real-time applications such as toxins, viruses, whole-cell, bacteria, nucleic acids, detection, or tissue impedance modeling [1,2,3,4,5,6]. Two-, three-, or four-electrode transductions are used for various impedance measurements. In general, the ratio between the voltage across the reference electrode (RE) and the working electrode (WE) and the current flowing between the working electrode and the counter electrode (CE) yields the impedance of a specific interface (solution electrode) [1,7] or tissue [8], cell culture [9], etc. The variable impedance of the transducer is generally estimated by AC electrochemical impedance spectroscopy (EIS) [1]. The EIS method measures the medium or interface’s impedance at multiple frequencies [10,11]. Next, proper fitting techniques estimate the impedance model from the spectrum [12]. However, for the development of a simple real-time, field-deployable readout interface, a single frequency AC excitation [1,3] is generally a good alternative for applications where the model of the interface or tissue is known a priori and the quantification of a specific analyte or change in the impedance due to bio-recognition is desired. One method to quantify the transducer impedance change is measuring the amplitude and phase of the response signal (current or voltage) with respect to a reference signal in real-time [13].

However, one challenge in the design of readout interfaces for label-free real-time single-frequency measurements is that the total change in the impedance of the transducers is often only a few percent over the entire full range of detection. For example, capacitive transducers for measuring certain hepatotoxins in water have been presented in [2], where the total electrode–solution interfacial capacitance changes by only 1% over the entire range of concentrations. A similar issue is associated with the developed label-free transducers for detecting Cholera toxins in [14] and Cryptosporidium at lower concentrations [15]. The impedance phase change at sub-Hz frequency is employed to detect human interleukin-8 in serum with sub-pg/mL sensitivity [16]. The results indicate a phase change of only $173\;m^{\circ }$ over a baseline impedance phase of $-86.6^{\circ }$, at the minimum detection level and an overall sensitivity of 220.4 m°/decade (approximately 0.25%). The resistance of the ultra-sensitive interdigitated electrodes designed in [17] changes by roughly a couple of percent over the full range of PfHRP2—a malaria biomarker—concentrations in human saliva. The non-faradaic EIS biosensor for detecting C-reactive protein in a complex medium, such as human blood, reports a fractional impedance change of approximately 2.5% at the minimum detection limit [18]. The capacitance of the developed DNA sensors changes by roughly 3% for the reported concentrations [19]. To complicate matters, avoiding physical damage to the functional layer and nonlinear distortion effect on the response require that the magnitude of the applied AC excitation remain small (typically <50 mV) [1,3,7]. The result is that the absolute value of the transducer output voltage is small, leading to a full-scale change in the voltage in the order of hundreds of microvolts. Moreover, suppose we assume that the transducer’s output voltage will be detected with a modest resolution of 8-bits. In that case, the sensor must be able to detect changes in the transducer’s output voltage that are less than 1 μV.

The readout process for the abovementioned sensitive transducers is straightforward, using laboratory-grade bench-top test equipment for characterization, but very sensitive instrumentation is required for large-scale deployment. As a solution, differential sensing with a bridge circuit that acts similar to intermediate secondary transduction and a sensitivity booster is proposed in [20]. Two capacitive transducer chips, each with a pair of electrodes (functionalized WE and chemically non-functional CE providing the electrical signal path in the solution), are placed in the opposite bridge legs and generate a differential sensing response. Although with the differential capacitive bridge designed in [20], the effects of common-mode noise, drift, and temperature variation can be further decreased and sensitivity enhanced, there is still a need for small, inexpensive, and low-power readout to reach a particular required resolution and make the commercialization of the developed transducers in this structure feasible [8,9].

**Figure 1:**
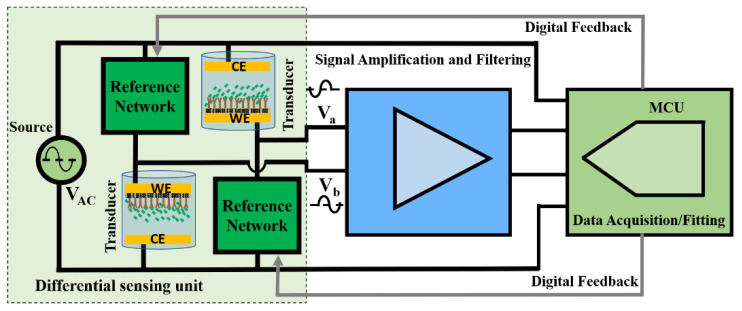
General block diagram of a differential bio-sensing unit interfaced with signal amplification and filtering unit and a microcontroller (MCU) for low-cost real-time operation and field deployment. The transducers are designed to sense the change in the transducer impedance due to bio-recognition with respect to the reference networks differentially. Amplification/filtering is typically required with the expected weak and noisy sensor response. For real-time and low-cost operation, an MCU performs both the task of data acquisition and processing (fitting) and any extra required digital adjustments.

Figure 1 shows the block diagram of the differential sensing unit utilized in [20] interfaced with high-level generic blocks of a readout system, amplification, filtering, and digitization. The transducers are designed to sense the change in the transducer impedance due to bio-recognition with respect to the reference networks differentially. Amplification/filtering is typically required with the expected weak and noisy sensor response. For real-time and low-cost operation, an MCU performs both the task of data acquisition and processing (fitting) and any extra required digital adjustments. The MCU acquires the response and excitation source signals and measures the amplified response signal magnitude and differential phase (with respect to the excitation source) in real-time.

Some factors limit the performance of the generic sensor interface units shown in Figure 1 if implemented with discrete commercially available components. Typical precision opamps that would be used in this application have input referred noise voltages that are on the order of tens of $\mathrm{nV}/\sqrt{\mathrm{Hz}}$. Therefore, to maintain a practical input signal-to-noise ratio (SNR) greater than 1, with microvolt-level transducer output voltages, the effective noise bandwidth of the sensor interface circuit could be limited to only a few tens of hertz. Moreover, approximately 60–80 dB voltage gains will be required to effectively use an ADC for data acquisition. Extreme amplification and filtering require highly accurate component matching, extensive shielding, and careful circuit design. Data acquisition and the specific algorithm implemented in the MCU for data processing in Figure 1 should be performed simultaneously with no need to transmit or store the raw data in external memory. Therefore, the algorithm processing speed and the ADC sampling rate should be carefully adjusted. Lower complexity, power consumption, and overall cost are other design goals to avoid limiting the potential of the sensor.

This paper proposes a comprehensive, fully differential readout interface design suitable for tiny sensor response signals generated differently. A study of the design tradeoffs between overall sensor system complexity and performance at low cost, particularly with small fractional detection impedance change (<1%), is given. Each readout interface block shown in Figure 1 was analyzed and designed based on the required resolution, overall gain, SNR, etc. It is shown here that extreme amplification and filtering requirements can be met with the careful design of a two-channel digital acquisition and processing (sine fitting) utilizing a single microcontroller. A theoretical design procedure and a practical discrete implementation example are presented here, targeting mainly real-time, low-cost operation for less than 1% full-scale change in the transducer’s output voltage, and an 8-bit characteristic change resolution. In Section 2, design methods considering the most important performance parameters, such as overall gain, bandwidth, SNR, common-mode rejection ratio (CMRR), sampling rate, signal fitting algorithm, and real-time implementation for each of the readout blocks; amplification, filtering, and data acquisition/processing are explained in detail. The implementation results, board characterization, and real-time operation and sensitivity verification are presented in Section 3.

## 2. Materials and Methods

A reasonable assumption for the differential sensing unit shown in Figure 1 is that less than a 1% full-scale response change with the bio-recognition event is expected before any amplification. The change in the transducer’s impedance ($Z_{t}$) has a linear relationship with the change in the medium’s concentration. Thus, the differential output voltage of the sensing unit is linearly related to the transducer fractional impedance change, and the source voltage (1) shows this linear relationship.

(1) $$V_{a}-V_{b}\approx \alpha \frac{\Delta Z_{t}}{Z_{t}}V_{AC},$$

where $V_{a}-V_{b},$ is the differential output voltage of the sensing unit, α is a proportionality constant, $\frac{\Delta Z_{t}}{Z_{t}},$ is the transducer fractional impedance change, and $V_{AC},$ is the AC excitation source voltage. 

Before discussing the amplification stage, it is mandatory to examine some typical values for the expected differential voltage. Let us consider a typical excitation signal (*V_AC_*) with an AC amplitude of 1–100 mV. Assuming the case with α=1, and $\frac{\Delta Z_{t}}{Z_{t}}<1\%,$ the maximum change in the amplitude of the bridge output will be less than 1 mV. If the target resolution for the sensor is set to the typical 8-bits, then the expected least-significant bit (LSB) of the response will be several microvolts. With the minimum response signal level knowledge, the proceeding amplification and filtering stages can be designed, and parameters such as required gain, matching, bandwidth, etc., can be decided. However, dealing with such small signals requires the careful consideration of parasitic, noise, and any unwanted interfering signals and trying to minimize such effects and achieve the dynamic target range; these design considerations are discussed in the following.

### 2.1. Amplification and Filtering Analysis

Two critical design parameters for the amplification and filtering unit will impact the accuracy and precision of a fully differential readout circuit for detecting tiny fractional changes at the output: common-mode-induced differential conversion and noise. The common mode rejection ratio (CMRR) criteria here is the amplification and filtering interface’s ability to reject the common mode output voltage (for example, an equal DC signal required to bias the differential transducers) and amplify the differential output voltage change due to the transducer impedance change with detection. Failing to reject the common mode at the output, considering the high required differential gain, will lead to an unpleasant common mode to differential conversion that not only gives rise to a false detection signal but also limits the dynamic range. Noise will affect the lower detection limit and shrink the dynamic range. Various sources causing common-mode to differential conversion and SNR degradation at the interface’s output before ADC are analyzed in the following section. Based on this analysis, parameters such as the gain of each stage for a cascaded design, effective noise bandwidth (ENB) of the filter, and the required matching sensitivity can be determined and based on the best achievable SNR at the board output, a potential suitable fitting algorithm in the data acquisition unit can be picked and designed.

#### 2.1.1. Common Mode to Differential Conversion

**Figure 2:**
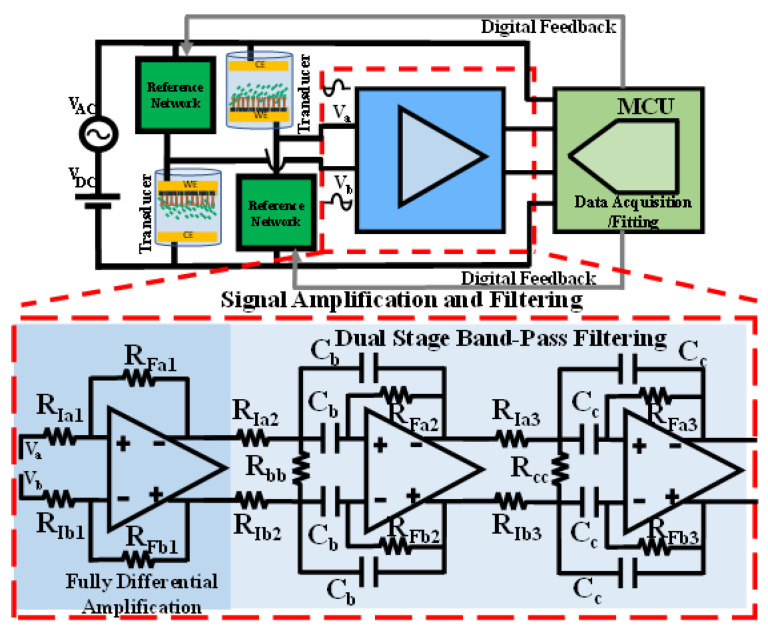
The three-stage amplification and filtering unit, proceeding the differential sensing unit and succeeding the data acquisition and fitting unit. A fully differential amplification followed by a cascaded two-stage bandpass filter provides the differential gain and filters the unwanted noise for certain minimum SNR. It is critical to maintain good matching in the feedback ratios of the peripheral components, as shown in (3), to achieve the overall target CMRR.

With the typical available full-scale voltage of the ADCs,$\;A_{FS}$, on the order of (1–5 V), a three-stage amplification and filtering interface is proposed here, as shown in Figure 2. One stage of amplification and two identical bandpass filtering stages are responsible for providing a total differential gain that amplifies the initial full-scale differentially sensed output of roughly 1 mV to match the full-scale range of the ADC. A multiple feedback bandpass topology is chosen for the fully differential filters [21]. The filter’s gain, center frequency, and bandwidth can be flexibly tuned with the multiple feedback topology. Therefore, a total differential gain in the order of 70–80 dB is approximately required. To estimate the amount of needed common mode rejection, consider the case with α=1/2 in (1). With $\left|V_{a}\right|=\left|V_{b}\right|=\left|V_{ab,CM}\right|$ equal to tens of millivolts (based on the excitation source amplitude <100 mV) and the differential LSB change at the sensing unit output, $\left|{\left(V_{a}-V_{b}\right)}_{LSB}\right|$, will only be several microvolts. 

$\left|V_{ab,CM}\right|$ and $\left|{\left(V_{a}-V_{b}\right)}_{LSB}\right|$ will be amplified by the common mode to differential gain and differential gain, respectively. To maintain the desired resolution, criteria (2) should be satisfied so that the differentially amplified sensing signal at the LSB level is at least twice the interference caused by the common mode to differential conversion:

(2) $$\frac{A_{v,diff}\cdot \left|{\left(V_{a}-V_{b}\right)}_{LSB}\right|}{A_{v,CMdiff}\cdot \left|V_{ab,CM}\right|}>2,$$

where $A_{v,CMdiff}$, is the common mode-induced differential gain, $A_{v,diff}$, is the differential gain. $\left|{\left(V_{a}-V_{b}\right)}_{LSB}\right|,$ and $\left|V_{ab,CM}\right|$ are the minimum differential voltage and the common mode voltage at the output of the sensing unit, respectively. The abovementioned criteria (2) sets the minimum for the $CMRR_{Total}=\frac{A_{v,diff}}{A_{v,CMdiff}}$, to around 100 dB which, with $A_{v,diff}$ of around 70–80 dB, requires the $A_{v,CMdiff}$ to be at least −30 to −20 dB. If the required minimum $CMRR_{total}$ is not reached, the target resolution will not be achieved.

- Effect of component matching and tolerance

Ideally, the CMRR of a fully differential circuit would be infinite. However, in reality, the CMRR is limited by the CMRR of each stage in the circuit and by matching the peripheral components. Typical fully differential amplifiers for precision measurements provide relatively high CMRR values of around 100 dB. However, when configured as a fully differential amplification stage, as shown in Figure 2, the CMRR of each stage is determined not only by the CMRR of the Opamp but also by the matching between the two symmetrical feedback ratios $\beta_{a}=\frac{R_{Fa1}}{R_{Fa1}+R_{Ia1}}$ and $\beta_{b}=\frac{R_{Fb1}}{R_{Fb1}+R_{Ib1}}$. If $\beta_{b}=\beta +\frac{\Delta \beta }{2}$ and $\beta_{a}=\beta -\frac{\Delta \beta }{2}$, therefore, $\beta_{b}-\beta_{a}=\;\Delta \beta$ and $\beta_{b}+\beta_{a}=2\beta$. The stage CMRR is derived in [22]:

(3) $${CMRR}_{Stage}\approx \frac{1}{\frac{1}{{CMRR}_{Opamp}}+\frac{\Delta \beta }{\beta }},$$

where ${CMRR}_{Stage},\;$ is the effective CMRR of each stage, ${CMRR}_{Opamp}$ is the typical CMRR of any given Opamp, and $\frac{\Delta \beta }{\beta },$ is the feedback matching ratio. Therefore, inevitable degradation in ${CMRR}_{Opamp}$ is expected with the worse matching ratio of the feedback networks.

For a given stage with a differential voltage gain, $A_{v,dd}$, $R_{Fb}=A_{v,dd}R_{Ib}$, the resistance, R’s tolerance ΔR, is included as $R_{Ib}=R\pm \Delta R,\;R_{Fb}=A_{v,dd}R\pm \Delta R$. Feedback ratio matching is related to the component tolerance using the following derivations:

(4) $$\frac{\Delta \beta }{\beta }=\frac{\beta_{b}-\beta_{a}}{\beta }=\frac{\frac{A_{v,dd}R\pm \Delta R}{\left(A_{v,dd}+1\right)R\pm 2\Delta R}-\frac{A_{v,dd}R\pm \Delta R}{\left(A_{v,dd}+1\right)R\pm 2\Delta R}}{\frac{A_{v,dd}R\pm \Delta R}{\left(A_{v,dd}+1\right)R\pm 2\Delta R}}=\frac{\pm 2\frac{\Delta R}{R}}{\left(A_{v,dd}+1\right)\pm 2\frac{\Delta R}{R}},\;$$

**Figure 3:**
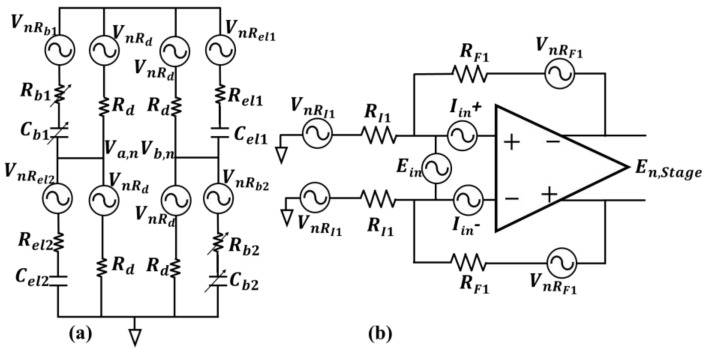
Utilized noise models for the two primary noise contributing blocks; (**a**) the bridge as the differential sensing unit, with the series RC model for the capacitive transducers and balancing networks, all the noise sources in the bridge are thermal type (**b**) differential amplification stage with the associated thermal noise sources for the peripheral resistors, and input-referred current and voltage noise sources of the Opamp.

For $\frac{\Delta \beta }{\beta }\ll 1$,

(5) $$\pm \frac{\Delta R}{R}=\frac{\Delta \beta }{\beta }\frac{\left(A_{v,dd}+1\right)}{2\left(1-\frac{\Delta \beta }{\beta }\right)}\approx \frac{\Delta \beta }{\beta }\frac{\left(A_{v,dd}+1\right)}{2},\;$$

where $\frac{\Delta R}{R},$ is the percent tolerance of a resistance. Based on (6), a lower component tolerance is required to maintain a given feedback-matching ratio for a higher differential gain of a stage. For the design and implementation of a multistage fully differential amplification unit, as shown in Figure 2; (3), and (5) determine the required degree of component matching tolerance based on the known differential gain distribution and total CMRR.

- Effect of cascaded stages

The abovementioned design criteria are regarding singular stages; now, given that several stages participate in the amplification and filtering before the data acquisition, it is helpful to understand each stage’s contribution to the total CMRR. In [23], the contribution of each stage, on the total CMRR, of a cascade of 3 differential stages is investigated, and an approximate formula, (6), is derived.

(6) $$\frac{1}{CMRR_{Total}}\approx \frac{1}{CMRR_{Stage_{1}}}+\frac{1}{\frac{A_{v,dd_{2}}}{A_{v,cc_{2}}}CMRR_{Satge_{2}}}+\frac{1}{\frac{A_{v,dd_{2}}}{A_{v,cc_{2}}}\;\frac{Av{,}_{dd_{3}}}{Av{,}_{cc_{3}}}CMRR_{Satge_{3}}},\;$$

where, $CMRR_{Total}$, is the total effective CMRR of the cascaded stages, $CMRR_{Stage}$ is the CMRR contribution of the individual amplification stage, $A_{v,dd}$, and $A_{v,cc}$ are the differential to differential and common mode to common mode gains of each of the 3 stages, respectively. If the output common-mode voltage,$\;V_{OCM}$, of the fully differential amplifiers before ADC, are set externally at $A_{FS}/2$, to cover the full ADC dynamic range equally, then the $A_{v,cc}$ for all stages would be 1. Thus, if the two filtering stages are identical, with the same gains, based on the (6) effect of the CMRR of the first stage on the $CMRR_{Total}$ is dominant compared to higher stages. Therefore, component matching in the first amplification stage significantly affects the total CMRR and common mode to differential conversion, and this is specifically important for implementation and debugging.

#### 2.1.2. Noise Analysis 

In addition to the common-mode-to-differential conversion, noise is also a primary limitation on the performance of the sensor readout circuitry. The presence of thermal noise, if the noise floor is greater than the detection signal around the lower detection limit, will decrease the dynamic range. Two primary sources of noise are involved in the general block diagram of the biosensor in Figure 1.; the total noise generated by the electrodes and differential sensing unit and noise generated by the amplification block. Quantization noise and sampling clock jitter are also issues, but the quantization noise effect is negligible if the utilized ADC resolution is higher than the required response target resolution. The clock jitter gains more importance at high sampling rates (hundreds of MS/s or GS/s) for higher frequency excitations; this effect will be considered and discussed separately in sub-Section 2.2.2.

Given the small magnitude of the electrode response on the order of several microvolts at LSB of detection, the noise of proceeding stages plays a vital role in determining the minimum expected SNR. The typical input referred voltage noise density for differential precision amplifiers is several $\frac{\mathrm{nV}}{\sqrt{\mathrm{Hz}}}$. If a gain of 70–80 dB is targeted utilizing the multistage amplification, the total output noise voltage density contributed by the amplifiers will be on the order of several $\frac{\mu V}{\sqrt{\mathrm{Hz}}}$. Moreover, the noise associated with the peripheral resistors and noise from the transducer itself will add to the total output noise density. Therefore, considering wide-band amplification stages without filtering, the minimum SNR value before ADC could obtain even less than 0 dB. However, the amount of in-band noise can be significantly reduced if bandpass filtering is performed. The quantitative analysis of the expected noise level presented in the following determines the method to calculate the required reduction in noise and effective noise bandwidth (ENB) of the filter.

The noise model of the electrodes and the equivalent circuitry with noise sources for the differential sensing unit and amplification stage are required for the noise analysis. The differential bridge model utilizing capacitive transducers presented in [20] is adopted here as the differential sensing unit. The noise model for the differential sensing unit with the noise sources included for noise analysis is shown in Figure 3a.

In Figure 3a, $R_{b1},\;C_{b1},R_{b2}\;$and $C_{b2}$ are the RC-balancing networks made with arrays of digitally controlled capacitors and resistors to balance the bridge for the AC signal path. $R_{d}$ resistors are DC-balancing resistive paths that provide a stable DC bias to the working electrodes and equal DC voltage at $V_{a}$ and $V_{b}$. The solution–electrode interface in this study is modeled with series RC, the resistive parts ($R_{el1}$ and $R_{el2}$) are modeling solution resistance and the capacitive parts ($C_{el1}$ and $C_{el2}$) are the interface capacitance. Gesteland et al. show in [24] that the noise of a metal microelectrode can be modeled as the thermal noise of a resistance in a narrow band of frequency, where the corresponding resistance is the real part of the electrode–solution interface impedance. Therefore, the associated noise sources with the electrodes and balancing resistors in the differential sensing unit is in series with the corresponding resistances in Figure 3a and are all representing thermal noise model for the resistance, i.e., $V_{nR}=\sqrt{4kTR},\;$with units of $V/\sqrt{\mathrm{Hz}}\;$. k is the Boltzmann’s constant, T is the absolute temperature and R is the corresponding resistance. For a perfectly matched and balanced bridge case, with $C_{el1}=C_{el2}=C_{b1}=C_{b2}=C_{X}$, $R_{el1}=R_{el2}=R_{b1}=R_{b2}=R_{X}$, the noise contribution of the differential sensing unit can be derived based on the model in Figure 3a as follows:

(7) $$E_{n,Bridge}^{2}=V_{a,n}^{2}+V_{b,n}^{2}=\frac{R_{d}^{2}C_{X}^{2}\omega_{in}^{2}}{1+{\left(R_{X}+R_{d}\right)}^{2}C_{X}^{2}\omega_{in}^{2}}V_{nR_{X}}^{2}+\frac{1+R_{X}^{2}C_{X}^{2}\omega_{in}^{2}}{1+{\left(R_{X}+R_{d}\right)}^{2}C_{X}^{2}\omega_{in}^{2}}V_{nR_{d}{}_{\;}}^{2},\;$$

where $E_{n,Bridge}^{2},\;$is the output referred noise power density of the bridge, and $\omega_{in}=2\pi f_{in}$, is the angular frequency of the operation. The electrodes makeup and solution conductivity for capacitive transducers are set so that at the frequency of operation, the reactive part of the interface impedance is dominant, $R_{el}<\left|1/jC_{el}\omega_{in}\right|\;\to R_{el}C_{el}\omega_{in}<1$ [1]. The value of the DC-balancing resistors, $R_{d}$, are much larger than the magnitude of the transducer impedance not to load the functional electrode impedance and decrease sensitivity, therefore, knowing that $R_{el}^{2}C_{el}^{2}\omega_{in}^{2}\ll 1$ leads to $R_{d}{}^{2}C_{el}^{2}\omega_{in}^{2}\gg 1$ [20].

Based on the abovementioned considerations, (7) can be simplified as follows:

(8) $$E_{n,Bridge}^{2}\approx \frac{R_{d}^{2}}{{\left(R_{X}+R_{d}\right)}^{2}}V_{nR_{X}}^{2}+\frac{1}{{\left(R_{X}+R_{d}\right)}^{2}}V_{nR_{d}{}_{\;}}^{2},\;$$

The noise model for a fully differential Opamp with the associated feedback resistors [25,26] is shown in Figure 3b. Again, the resistors have the thermal noise voltage model in series, and $E_{in}$, $I_{in+}$ and $I_{in-}$ are the Opamp input referred voltage and current noise sources, respectively. The power density of the Opamp noise sources have the units of $V^{2}/\mathrm{Hz}$ and are defined as; $E_{in_{i}}^{2}=e_{w}^{2}\left(1+\frac{f_{enc}}{f}\right)$, $I_{in_{i}-}^{2}=I_{in_{i}+}^{2}=i_{w}^{2}\left(1+\frac{f_{inc}}{f}\right)$. $e_{w}^{2}$ and $i_{w}^{2}$, are the opamp input referred voltage and current white noise powers. $f_{enc}$ and $f_{inc}$, are the voltage and current noise power density corner frequencies. The output referred noise power density of each amplification and filtering stage is $E_{n,Stage_{i}}^{2}$, For the case of a noiseless excitation source, the total root mean square (rms) noise voltage at the output of the amplification and filtering board is obtained from (9).

(9) $$\bar{E_{n,Total}}=\sqrt{\int_{f_{L}}^{f_{H}}(G_{N_{1}}^{2}G_{N_{2}}^{2}G_{N_{3}}^{2}E_{n,Bridge}^{2}+G_{N_{2}}^{2}G_{N_{3}}^{2}E_{n,Stage_{1}}^{2}+G_{N_{3}}^{2}E_{n,Stage_{2}}^{2}+E_{n,Stage_{3}}^{2})df\;},\;$$

where $\bar{E_{n,Total}}$ is the total rms noise voltage at the board output. Here, it is also assumed that the output common mode voltage is set externally and adequately filtered. The output referred noise power density of the bridge, $E_{n,Bridge}^{2}$, and the output referred noise power density of each amplification and filtering stage, $E_{n,Stage_{i}}^{2}$, is multiplied by the square of the noise gain, $G_{N_{i}}=1+\frac{R_{F}{}_{i}}{R_{I_{i}}}$ of the proceeding stages, then summed and integrated over the bandwidth of the interface (lower, $f_{L}$, to higher, $f_{H}$, 3 dB cut off frequency). The output referred noise power density of each differential amplification and filtering stage is obtained from (10).

(10) $$E_{n,Stage_{i}}^{2}=G_{N_{i}}^{2}E_{in_{i}}^{2}+R_{F_{i}}^{2}\left(I_{in_{i}-}^{2}+I_{in_{i}+}^{2}\right)+2\;V_{n_{R_{F_{i}}}}^{2}+2{\left(\frac{R_{F_{i}}}{R_{I}{}_{i}}\right)}^{2}V_{n_{R_{I_{i}}}}^{2},\;$$

where $V_{nR_{X}}^{2}=4kTR_{X}$, and $V_{nR_{d}}^{2}=4kTR_{d}$. Assuming $\frac{f_{H}-f_{L}}{f_{L}}<1$, $\frac{1}{f_{in}^{2}}\ll 1$, $\frac{f_{enc}}{f_{L}}\ll 1$ and $\frac{f_{inc}}{f_{L}}\ll 1$, yields (11). For a 4th-order bandpass filter (consisting of two stages in this design) $ENB=1.025\left(f_{H}-f_{L}\right)\;$ [25,27] Note that if $f_{enc}>f_{L}$ and $f_{inc}>f_{L}$, the effect of flicker noise must be included, as well. Based on (11), the ENB of the required overall filtering to obtain 8-bit resolution at a given expected SNR can be determined using the typical application values for the bridge components.

(11) $$\bar{E_{n,Total}}\approx \sqrt{ENB}\times \sqrt{\begin{array}{c} G_{N_{1}}^{2}G_{N_{2}}^{2}G_{N_{3}}^{2}(V_{n_{R_{X}}}^{2}{\left(\frac{R_{d}}{R_{d}+R_{X}}\right)}^{2}+V_{n_{R_{D}}}^{2}{\left(\frac{1}{R_{d}+R_{X}}\right)}^{2})+\\ 2G_{N_{2}}^{2}G_{N_{3}}^{2}V_{n_{R_{F_{1}}}}^{2}+2G_{N_{2}}^{2}G_{N_{3}}^{2}{\left(\frac{R_{F_{1}}}{R_{I}{}_{1}}\right)}^{2}V_{n_{R_{I1}}}^{2}+\\ e_{w}^{2}\left(G_{N_{1}}^{2}G_{N_{2}}^{2}G_{N_{3}}^{2}+G_{N_{2}}^{2}G_{N_{3}}^{2}+G_{N_{3}}^{2}\right)+i_{w}^{2}\left(2G_{N_{2}}^{2}G_{N_{3}}^{2}R_{F_{1}}^{2}+2G_{N_{3}}^{2}R_{F_{2}}^{2}+2R_{F_{3}}^{2}\right)+\\ 2G_{N_{3}}^{2}V_{n_{R_{F_{2}}}}^{2}+2G_{N_{3}}^{2}{\left(\frac{R_{F_{2}}}{R_{I}{}_{2}}\right)}^{2}V_{n_{R_{I2}}}^{2}+2V_{n_{R_{F_{3}}}}^{2}+2{\left(\frac{R_{F_{3}}}{R_{I}{}_{3}}\right)}^{2}V_{n_{R_{I3}}}^{2} \end{array}}$$

Thus, the minimum expected SNR is computed by knowing the minimum expected rms differentially sensed and amplified signal with a defined resolution and the total rms noise signal after filtering. The required ENB can be adjusted accordingly.

(12) $$SNR_{min}=20\times \log\left(\frac{A_{v,diff}\cdot \left|{\left(V_{a}-V_{b}\right)}_{LSB,rms}\right|}{\bar{E_{n,Total}}}\right),\;$$

where $A_{v,diff}\cdot \left|{\left(V_{a}-V_{b}\right)}_{LSB,rms}\right|$ is the minimum expected rms differentially sensed and amplified signal for a given resolution. In practice, however, for low-frequency (<10 kHz) measurements, the effect of flicker noise cannot be neglected entirely, and other sources of non-ideality, such as the transducer functional layer instability and environmental noise, may also add to the estimated total output referred rms noise, and even lower ENB might be required. There are also practical limitations on realizing filter bandwidth on the order of 10s of hertz. The minimal bandwidth leads to a longer response settling time for step-type input variations. Sometimes the dynamics of analyte binding are fast, and the readout interface should be able to follow the relatively fast variations in the response signal. Therefore, the bandwidth should be set considering the amount of allowable noise and the fast settling requirement. Although a higher filter bandwidth leads to worse SNR, proper further digital signal processing techniques with suitable sine-fitting algorithms can effectively act as an additional filter and even extract the signal information buried in the noise, and this is described next.

### 2.2. Real-Time Digitization and Fitting

**Figure 4:**
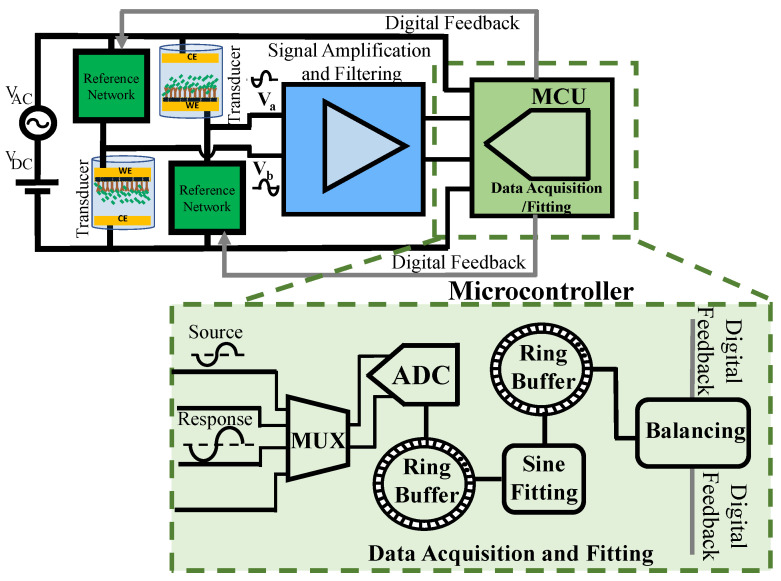
A single microcontroller is utilized to both digitize the source and response signals. The built-in ADC samples the source and response signals alternatively. The samples are stored in ring buffers, and MCU computes the amplitude and differential phase information. The results are stored in a ring buffer for external communication.

For any biosensor’s deployment, the transducer’s characteristic change should ultimately be quantified. In general, the response is an electric DC or AC signal that needs to be acquired in real-time for field deployment. The complex impedance of the solution–electrode interface can be measured in real-time by extracting the amplitude and phase data from the amplified transducer’s response signal. While amplitude information can be obtained using the response signal, the phase should be measured differentially with respect to some reference signal. In the two-channel data acquisition system, shown in Figure 4, the ADC alternatively samples the excitation voltage (i.e., the source signal) as a reference for differential phase measurement and the output of the amplification/filtering block (i.e., the response signal). To automate this, the digital system must be able to extract the amplitude and differential phase quantities from the digitized signal using a specific algorithm and in real-time for field deployment. Simultaneous data acquisition and real-time processing become feasible using ring buffers without sacrificing memory. The ADC samples the source and response signals and writes the data into the designated ring buffer. A sine fitting algorithm implemented within the microcontroller reads the samples consecutively and applies the fitting algorithm to a specific number of samples as a batch.

The fitting results containing the amplitude and differential phase information are written in another ring buffer. The MCU uses the results for balancing-related computations or external communication via UART. The key parameters for the data acquisition and fitting unit design are the ring buffer length, ADC sampling rate, type of the sine fitting algorithm, and the number of samples required for each round of fitting, considering the expected signal and noise levels. Additionally, as the processing occurs in real-time using a single MCU, it requires a computationally efficient code and algorithm. The design process is explained in the following.

Sine-fitting algorithms are traditionally seen in ADC testing and characterization [28] and impedance/frequency response measurements [29].

Researchers have recently recognized the utility of sine-fitting algorithms for the real-time processing of the transducer output voltage [29,30]. There are many different approaches to sine-fitting, each with varying degrees of suitability in low-cost, real-time sensing applications. To design an accurate real-time sine fitting, the designer must set many parameters such as sampling rate, SNR, ADC resolution, record length, etc. Unfortunately, there is no general study of important factors for sine fitting of biosensor response in real-time; thus, the designer must study these tradeoffs for each design. This section will present a detailed analysis of the various tradeoffs for the abovementioned design parameters concerning sensing requirements. The results will help the designer pick the proper sine fitting algorithm based on the available budget, expected noise floor, required dynamic range, and accuracy with specific DSP hardware capabilities.

In general, sine-fitting algorithms can be classified as either iterative or non-iterative. Although providing better accuracy in some applications, iterative algorithms, such as the IEEE standard 4-parameter sine fitting [28], are not the best candidates for real-time implementation. By nature, the convergence of these algorithms might require multiple iterations, and data storage requires additional memory usage. Non-iterative algorithms, on the other hand, offer better real-time solutions, considering their relatively more straightforward implementation. However, the accuracy of a non-iterative approach with small and noisy signals and compatibility with low-cost general-purpose microcontroller implementation needs to be considered for real-time and field deployable applications.

The non-iterative sine parameter extraction algorithm, IEEE standard 3-parameter sine fit (3PSF) [31], is reviewed for the design requirements in this paper. 

The 3-parameter sine parameter extraction is based on the assumption that the excitation source frequency ($f_{in}$) is known, and only the amplitude, initial phase, and DC offset of each singular channel are estimated. A brief theoretical review of the 3PSF is available in [31,32,33]. The real-time implementation of 3PSF in a low-cost microcontroller can be greatly simplified by using coherent sampling; therefore, simplified 3PSF with coherent sampling is considered for performance comparison here. Consider a sequence of N samples $\left(k=0,\;1,\ldots N-1\right)$ of a sine wave represented as follows:

(13) $$y\left[k\right]=A\cos\left(2\pi \frac{f_{in}}{f_{s}}k+\varphi \right)+DC.$$

where $f_{s}$ is the sampling rate for both channels (source and response), sine wave frequency $f_{in}$, amplitude A, initial phase φ, and offset DC. The source and response signals are derived from the same generator; therefore, they share the same frequency $f_{in}$. If the ratio of $f_{in}/f_{s}$ is known, the 3 parameters A, φ, and DC can be estimated for each channel, in the least squares sense, using 3PFS, as shown in [31]. The real-time implementation of 3PSF in a low-cost microcontroller can be significantly simplified by using coherent sampling; therefore, simplified 3PSF with coherent sampling is considered for performance comparison here. 

If the estimated signal, $\hat{y}\left[k\right]$ is expressed as follows:

(14) $$\hat{y}\left[k\right]={\hat{A}}_{c}\cos\left(2\pi \frac{f_{in}\;}{f_{s}\;}k\right)+{\hat{A}}_{s}\sin\left(2\pi \frac{f_{in}\;}{f_{s}\;}k\right)+\hat{DC}$$

With some simple algebra using the coefficients ${\hat{A}}_{c}$ and ${\hat{A}}_{s}$ for each channel, the amplitude and phase of y are estimated as in [31].

(15) $$\hat{A}=\sqrt{{\hat{A}}_{c}^{2}+{\hat{A}}_{s}^{2}}$$

(16) $$\hat{\varphi }=\tan^{-1}\left(\frac{-{\hat{A}}_{s}}{{\hat{A}}_{c}}\right).$$

where $\hat{A}$ and $\hat{\varphi }$ are the estimated amplitude and initial phase. Coherent sampling is achieved when $f_{in}/f_{s}=M/N$, where M and N are relatively prime integers and represent the total number of input periods in the record and the total record length, respectively. Under the assumption of coherent sampling, ${\hat{A}}_{c}$, ${\hat{A}}_{s}$ and $\hat{DC}$ are defined as follows:

(17) $$\left[\begin{array}{c} {\hat{A}}_{c}\\ {\hat{A}}_{s}\\ \hat{DC} \end{array}\right]=\left[\begin{array}{c} 2/N\overset{N-1}{\underset{k=0}{\sum }}y\left[k\right]\cos\left(2\pi \frac{M\;}{N\;}k\right)\\ 2/N\overset{N-1}{\underset{k=0}{\sum }}y\left[k\right]\sin\left(2\pi \frac{M\;}{N\;}k\right)\\ 1/N\overset{N-1}{\underset{k=0}{\sum }}y\left[k\right] \end{array}\right].\;$$

For a known input frequency, the given ratio of $f_{in}/f_{s}$ and the ratio of M/N remains fixed. Hence, a lookup table rather than a function can be used to compute the sinusoidal values, $\cos(2\pi \frac{M\;}{N\;}k$) and $\sin\left(2\pi \frac{M\;}{N\;}k\right)$, dramatically reducing the required processing time. A primary concern, however, remains the effect of uncertainty in the $f_{in}/f_{s}$ ratio, as well as jitter in the sampling clock, in the presence of very low SNR signals. Therefore, the effects of the oversampling ratio of the ADC and the SNR of the signal, as well as clock jitter and computational resource requirements, will be studied before algorithm implementation. The principal investigated performance metrics in the analysis and comparisons are the estimated percent amplitude error ($\epsilon_{A}$), and percent differential phase ($\varphi_{diff}$ = $\varphi_{response}-\varphi_{source}$) error ($\epsilon_{\varphi_{diff}}$). 

#### 2.2.1. Additive White Gaussian Noise

To see the effect of noise on the resolution of the sensor, let us assume the noise, $n_{k}$, is additive white Gaussian noise. For a noisy, coherently sampled signal,

(18) $$y_{\;noisy}\left[k\right]=A\cos\left(2\pi \frac{f_{in}\;}{f_{s}\;}k+\varphi \right)+DC+n_{k},\;$$

the estimation parameters in (17) are independent and unbiased in the presence of white Gaussian noise. In this case, the expected values of the estimation parameters are: $E\left[{\hat{A}}_{c}\right]={\tilde{A}}_{c}=A\cos\varphi$, $E\left[{\hat{A}}_{s}\right]={\tilde{A}}_{s}=A\sin\varphi$, and $E\left[\hat{DC}\right]=\tilde{DC}=DC$. The covariance matrix, $C_{noise}$, of the 3PSF with coherent sampling is [32]:

(19) $$C_{noise}=\left[\begin{array}{ccc} \frac{2}{N}\sigma_{n}^{2} & 0 & 0\\ 0 & \frac{2}{N}\sigma_{n}^{2} & 0\\ 0 & 0 & \frac{1}{N}\sigma_{n}^{2} \end{array}\right].$$

where $\sigma_{n}^{2}$ is the variance in the additive white Gaussian noise. The estimated amplitude parameters, ${\hat{A}}_{c}$ and ${\hat{A}}_{s}$, in the presence of noise, are statistically analyzed and defined in [32,33], with their expected values ${\tilde{A}}_{c}$ and ${\tilde{A}}_{s}$, and equal variance $2\sigma_{n}^{2}/N$. The amplitude and initial phase $\hat{A}$ and $\hat{\varphi }$, are functions of the statistically defined random variables ${\hat{A}}_{c}$ and ${\hat{A}}_{s}$. The mean and variance of a function of two random variables $f\left({\hat{A}}_{c},{\hat{A}}_{s}\right)$, can be approximately derived based on a Taylor series expansion of the function about the expected values of the associated random variables ${\tilde{A}}_{c},\;{\tilde{A}}_{s}$, as shown in [32,34]:

(20) $$E\left[f\left({\hat{A}}_{c},{\hat{A}}_{s}\right)\right]\approx f\left({\tilde{A}}_{c},\;{\tilde{A}}_{s}\right)+\frac{1}{2}\left(\frac{\partial^{2}f}{\partial {\hat{A}}_{c}{}^{2}}C_{11}+2\frac{\partial^{2}f}{\partial {\hat{A}}_{c}\partial {\hat{A}}_{s}}C_{12}+\frac{\partial^{2}f}{\partial {\hat{A}}_{s}{}^{2}}C_{22}\right),$$

(21) $$var\left[f\left({\hat{A}}_{c},{\hat{A}}_{s}\right)\right]\approx {\left(\frac{\partial f}{\partial {\hat{A}}_{c}}\right)}^{2}C_{11}+2\frac{\partial f}{\partial {\hat{A}}_{c}}\frac{\partial f}{\partial {\hat{A}}_{s}}C_{12}+{\left(\frac{\partial f}{\partial {\hat{A}}_{s}}\right)}^{2}C_{22},$$

where $E\left[f\left({\hat{A}}_{c},{\hat{A}}_{s}\right)\right]$ and $var\left[f\left({\hat{A}}_{c},{\hat{A}}_{s}\right)\right]$ are the mean and variance of the function $f\left({\hat{A}}_{c},{\hat{A}}_{s}\right)$. Using (19)–(21), the mean and variance of the estimated amplitude and initial phase can be derived as a function of SNR:

(22) $$E\left[\hat{A}\right]\approx \tilde{A}+\frac{1}{N}\frac{\sigma_{n}^{2}}{\tilde{A}}\approx \tilde{A}\left(1+\frac{1}{2N\cdot SNR}\right),\;var\left(\hat{A}\right)\approx \frac{2}{N}\sigma_{n}^{2}$$

(23) $$E\left[\hat{\varphi }\right]\approx \tilde{\varphi },\;var\left(\hat{\varphi }\right)\approx \frac{1}{N\cdot SNR}$$

where $E\left[\hat{A}\right]$, $var\left[\hat{A}\right]$ and $E\left[\hat{\varphi }\right]$, $var\left[\hat{\varphi }\right]$ are the mean and variance of the estimated amplitude and initial phase, respectively. Based on (22), the amplitude estimation is biased with noise present. When alternate sampling is used, as shown in Figure 4, the initial phase of the response signal is measured with reference to the source signal, therefore:

(24) $$var\left[{\hat{\varphi }}_{diff}\right]=var\left[{\hat{\varphi }}_{response}\right]+var\left[{\hat{\varphi }}_{source}\right]-2cov\left({\hat{\varphi }}_{response},{\hat{\varphi }}_{source}\right),\;$$

where $var\left[{\hat{\varphi }}_{diff}\right]$ is the variance of the differential phase, and $var\left[{\hat{\varphi }}_{response}\right]\;\mathrm{and}\;var\left[{\hat{\varphi }}_{source}\right]\;$are the variance of the initial phase estimation of response and source signals, respectively. When the source and response signals are corrupted by additive noise, the initial phase estimations are independent (i.e., $cov\left({\hat{\varphi }}_{response},{\hat{\varphi }}_{source}\right)$ = 0). From (23) and (24), if the same record length is assumed for both channels (*N*), the variance of the differential phase can be derived as:

(25) $$var\left[{\hat{\varphi }}_{diff}\right]\approx \frac{1}{N}\left(\frac{1}{SNR_{response}}+\frac{1}{SNR_{source}}\right)$$

The 3PSF algorithm can effectively reduce the effect of noise on the acquired data based on (22)–(25). If the ratio of M/N is chosen considering the actual SNR at the lower limits of detection, better resolution can be achieved. 

#### 2.2.2. Sampling Clock Jitter 

Sampling clock jitter causes uncertainty in 3PFS estimation results. The uncertainty caused by sampling clock jitter can be modeled as a normally distributed random variable $\alpha_{k}\;$with zero mean and standard deviation equal to $\sigma_{\alpha }$ [35]. A coherently sampled signal with jitter is modeled as follows:

(26) $$y_{jitter}\left[k\right]=A\cos\left(2\pi f_{in}\left(t_{k}+\alpha_{k}\right)+\varphi \right)+DC=A\cos\left(2\pi f_{in}t_{k}+\theta_{k}+\varphi \right)+DC,\;$$

where $\theta_{k}$ is a normally distributed random variable with zero mean and standard deviation $2\pi f_{in}\sigma_{\alpha }=\sigma$. In the presence of jitter, the 3PSF is no longer an unbiased estimator for ${\hat{A}}_{c}$ and ${\hat{A}}_{s}$, the expected values for the three parameters are as follows:

(27) $$E\left[{\hat{A}}_{c}\right]=Ae^{\frac{-\sigma^{2}}{2}}\cos\varphi ,\;$$

(28) $$E\left[{\hat{A}}_{s}\right]=-Ae^{\frac{-\sigma^{2}}{2}}\sin\varphi ,\;$$

(29) $$E\left[\hat{DC}\right]=DC,$$

(30) $$C_{jitter}=\left[\begin{array}{ccc} \frac{A^{2}}{N}\left(1-e^{-\sigma^{2}}\right)\left(1-\frac{1}{2}e^{-\sigma^{2}}\cos2\varphi \right) & 0 & 0\\ 0 & \frac{A^{2}}{N}\left(1-e^{-\sigma^{2}}\right)\left(1+\frac{1}{2}e^{-\sigma^{2}}\cos2\varphi \right) & 0\\ 0 & 0 & \frac{A^{2}}{N}\left(1-\frac{1}{2}e^{-\sigma^{2}}\right) \end{array}\right],\;$$

If the initial phase, φ, is assumed to be constant, the covariance matrix of the estimator in the presence of jitter, $C_{jitter}$, is derived in (30). The approximate mean and variance of the amplitude and initial phase estimation with jitter present, using (27) and (28), are as follows:

(31) $$E\left[\hat{A}\right]\approx Ae^{\frac{-\sigma^{2}}{2}}+\frac{A}{2N}\left(1-e^{-\sigma^{2}}\right)\left(e^{\frac{\sigma^{2}}{2}}+\frac{1}{2}e^{\frac{-\sigma^{2}}{2}}\right),\;$$

(32) $$var\left(\hat{A}\right)\approx \frac{A^{2}}{N}\left(1-e^{-\sigma^{2}}\right)\left(1-\frac{1}{2}e^{\frac{-\sigma^{2}}{2}}\right),$$

(33) $$E\left[\hat{\varphi }\right]\approx \tilde{\varphi }\;,\;var\left(\hat{\varphi }\right)\approx \frac{1}{N}\left(1-e^{-\sigma^{2}}\right)\left(e^{\sigma^{2}}+\frac{1}{2}\right),$$

In the presence of jitter, the estimations for the source and output initial phases are independent, i.e., $cov\left({\hat{\varphi }}_{response},{\hat{\varphi }}_{source}\right)=0$. The differential phase variance is derived using (24) and (33):

(34) $$var\left[{\hat{\varphi }}_{diff}\right]\approx \frac{1}{N}\{\left(1-e^{-\sigma_{source}{}^{2}}\right)(e^{\sigma_{source}{}^{2}}+\frac{1}{2})+\left(1-e^{-\sigma_{response}{}^{2}}\right)(e^{\sigma_{response}{}^{2}}+\frac{1}{2})\},\;$$

If we examine the mean error in the estimated amplitude using (31) from above:

(35) $$\underset{N\to \infty }{\lim}\varepsilon_{A}=\frac{E\left[\hat{A}\right]-A}{A}=e^{\frac{-\sigma^{2}}{2}}-1,$$

where $\varepsilon_{A}$ is the amplitude estimation relative error. Although it is seen in (35) that the amplitude mean error will never reach zero even with the largest number of samples for 3PSF, which is also claimed in [35], with an optimized large record length 8-bit detection resolution for both amplitude and phase is achievable even at non-realistically high jitter standard deviation of around π radians.

Based on the obtained results, 3PSF can maintain the target 8-bit resolution at jitter standard deviations, even close to 2π radians, by controlling the record length.

It is worth mentioning that the obtained result for this analysis depends on the source and sampling frequency. The maximum allowable jitter using an ADC with a resolution of R bits, and a sine wave input with an amplitude equal to the ADC full-scale and a frequency of $f_{in}$, to have a jitter-induced error of less than half LSB is inversely proportional to ($2\pi f_{in}2^{R})$ [36]. The maximum allowable jitter, therefore, grows smaller if the sine wave has an amplitude smaller than full-scale, higher frequency, and with higher resolution for the ADC. For example, jitter considerations gain more importance for sensors with hundreds of MHz or GHz level excitation frequencies or when the response signal is not sufficiently amplified to the ADC’s full-scale range at lower detection limits.

#### 2.2.3. Non-Coherency

A fundamental assumption while simplifying the implementation of the real-time 3PSF algorithm is that the data belong to a coherently sampled sine wave. However, depending on the source sine wave generator’s accuracy level, the desired source frequency may deviate from its actual value. The result will be that the record will not contain exactly M cycles of the input signal, and the readily hard-coded lookup table for computing the sinusoidal values, $\cos(2\pi \frac{M}{N}k$) and $\sin\left(2\pi \frac{M}{N}k\right)$. will no longer represent correct samples, leading to errors in the estimated amplitude and initial phase. The effect of a shift in the source frequency, Δf, can be modeled by assuming a shift in the $f_{in}$ such that [32]:

(36) $$\Delta f=f-f_{in}=\frac{\left(Q\cdot \delta \right)f_{s}}{N},$$

where Q is the integer part, and δ is the fractional part of the residue. The result is a shift in the number of periods being sampled. Now, if the cycles in the presence of shift in $f_{in}$, are $M^{\prime }=M+Q\cdot \delta$, the actual waveform, can be expressed as follows:

(37) $$y_{dev}\left[k\right]=A\cos\left(2\pi \frac{M+Q\cdot \delta }{N}k+\varphi \right),$$

where $y_{dev}\left[k\right]\;$are the samples of the waveform, including a frequency deviation. Using (15) and (17), the amplitude is estimated as follows:

(38) $$\hat{A}\left(Q\cdot \delta \right)={\left\{{\left[\frac{2}{N\sum_{k=0}^{N-1}A\cos\left(2\pi \frac{M+Q\cdot \delta }{N}k+\varphi \right)\cos\left(2\pi \frac{M\;}{N\;}k\right)}\right]}^{2}+{\left[2/N\sum_{k=0}^{N-1}A\cos\left(2\pi \frac{M+Q\cdot \delta }{N}k+\varphi \right)\sin\left(2\pi \frac{M\;}{N\;}k\right)\right]}^{2}\right\}}^{1/2}$$

which can be further simplified as a function of ∆f to be:

(39) $$\hat{A}\left(∆f\right)=A{\left[{\sin c}^{2}\left(N\frac{∆f}{f_{s}}+2M\right)+{\sin c}^{2}\left(N\frac{∆f}{f_{s}}\right)+2\sin c\left(N\frac{∆f}{f_{s}}+2M\right)\sin c\left(N\frac{∆f}{f_{s}}\right)\cos\left(2\varphi +2\pi N\frac{∆f}{f_{s}}\right)\right]}^{1/2},\;$$

The initial phase estimation, as a function of Q·δ, can be derived using (16) and (17):

(40) $$\hat{\varphi }\left(Q\cdot \delta \right)=\tan^{-1}\left(\frac{-2/N\sum_{k=0}^{N-1}A\cos\left(2\pi \frac{M+Q\cdot \delta }{N}k+\varphi \right)\sin\left(2\pi \frac{M\;}{N\;}k\right)}{2/N\sum_{k=0}^{N-1}A\cos\left(2\pi \frac{M+Q\cdot \delta }{N}k+\varphi \right)\cos\left(2\pi \frac{M}{N}k\right)}\right)$$

which can be further simplified and written as a function of frequency deviation, ∆f:

(41) $$\hat{\varphi }\left(∆f\right)=\tan^{-1}\left[\tan\left(\varphi +\pi N\frac{∆f}{f_{s}}\right)\frac{\tan\left(\pi \frac{f_{in}\;}{f_{s}\;}\right)}{\tan\left(\pi \frac{∆f+f_{in}}{f_{s}}\right)}\right],\;$$

With the realistic assumption that the shift in the input frequency is much smaller than the sampling frequency ($\Delta f\ll f_{S}$), (39) reveals that the amplitude estimation accuracy is highly dependent on the record length N. The larger the record length, the greater the amplitude estimation error for a given Δf. On the other hand, if $\Delta f\ll f_{S}$, (41) simplifies as follows:

(42) $$\hat{\varphi }\left(∆f\right)\approx \varphi +\pi N\frac{∆f}{f_{s}},\;$$

(42) indicates that, with a shift in the input frequency, regardless of the size of the sample record, there will be a linear phase error associated with the phase estimation. This error can be effectively mitigated if the output signal phase is measured with respect to a signal that shares the same frequency generator with the response. 

#### 2.2.4. Real-Time Microcontroller Implementation Considerations

For real-time implementation, any algorithm must perform mathematical operations with the individually taken samples from each channel, store the results in specific variables, and regularly update them with each incoming sample. The implementation cost is, therefore, the number of mathematical operations, functions, lookup tables, and the number of memory positions to hold the variables per fixed amount of data within each recording. One can estimate the execution time based on the number of clock cycles required by a specific MCU for each math operation. For the simplified 3PSF with a known excitation frequency, the values of $\sin\left(2\pi f_{in}t_{k}\right)$ and $\cos\left(2\pi f_{in}t_{k}\right)$ can be pre-computed using a singular lookup table. For comparison, the non-iterative ellipse fit algorithm [37] requires additional matrix operations other than the trigonometric functions and square root calculations that could be implemented with lookup tables. The real-time 3PSF is efficiently implemented within an MCU, with the computational cost as low as six variables, four multiplications, six additions, and one lookup table. 

## 3. Results

Provided the theoretical analysis of the critical factors limiting the performance of a fully differential amplification/filtering and data acquisition/processing board, a readout interface is designed, fabricated, and tested for target 8-bit impedance sensing resolution. Board implementation and performance verification procedures are discussed in this section. 

### 3.1. Differential Amplification and Filtering Implementation

**Figure 5:**
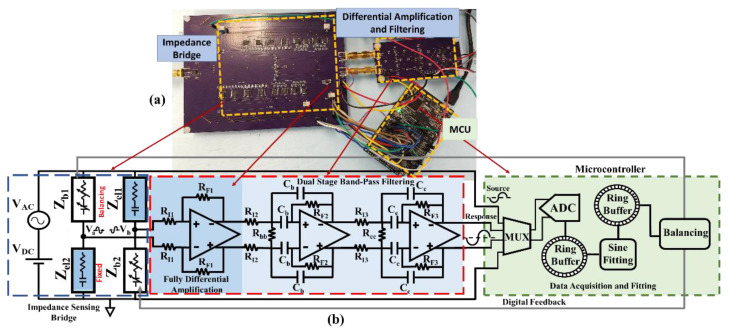
(**a**) The implemented amplification and filtering board interfaced with the differential impedance sensing unit: (**b**) The block diagram of the differential impedance sensing unit and the developed readout. The first amplification stage is placed on the same board as the sensing unit to avoid interferences with the small sensing response signal caused by external connections.

The Block diagram and the implemented boards of the amplification and filtering units interfacing a differential impedance sensing bridge are shown in Figure 5. A custom differential impedance sensing bridge with fixed series RC impedances ($Z_{el1}$ and $Z_{el2}$) mimicking the electrode–solution interface impedance and balancing networks ($Z_{b1}$ and $Z_{b2}$) composed of a digitally tunable resistor and capacitor array [20] is interfaced with the designed readout for performance verification. The DC biasing resistors ($R_{d}=50\;k\Omega$) are not shown on the schematic for simplicity but are included on the board, as shown in Figure 3a. The first fully differential amplification stage is fabricated on the same board as the differential sensing unit to minimize the interferences caused by wiring and external connections. Details about the part selection and component values using the proposed design methodology are explained in the next section.

#### 3.1.1. Effect of Component Matching and Tolerances

**Figure 6:**
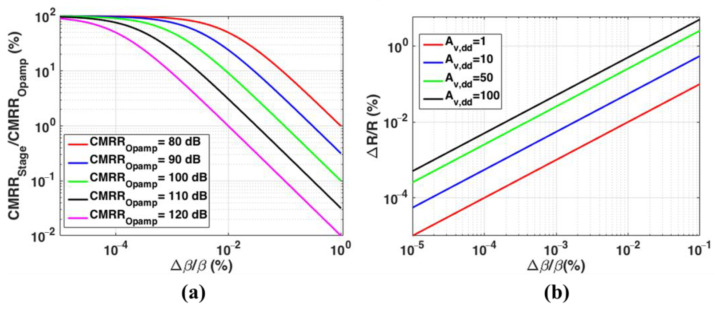
(**a**) Percent degradation in the ratio of the CMRR of a fully differential stage ($CMRR_{Stage}$) and Opamp typical CMRR ($CMRR_{Opamp}$) for various $CMRR_{Opamp},\;$versus percent change in matching ratios of peripheral resistors constructing the feedback network (Δβ/β); (**b**) Percent resistance tolerance (ΔR/R) for various differential stage gain, $A_{v,dd}$, versus percent feedback ratio mismatch (Δβ/β).

The three-stage amplification is configured to provide a total differential gain of 70 dB, to amplify the sensing unit’s full-scale output to $A_{FS}$ of the ADC. The differential precision amplifier, Analog Devices Inc., Wilmington, MA, USA, LTC6363 is picked as the critical component of the amplification and filtering unit. The typical $CMRR_{Opamp}$, for LTC6363 is 110 dB. To maintain a total CMRR of around 100 dB for the readout, a common mode-induced differential gain of around −30 dB is required based on (2). Figure 6a shows the percent degradation in $CMRR_{Stage}/CMRR_{Opamp}$ for various $CMRR_{Opamp}$, versus the percent feedback matching ratio obtained from (3). Given a typical value of $CMRR_{Opamp}=110\;\mathrm{dB}$, feedback matching ratios better than 0.001% are required to obtain a $CMRR_{Stage}$ of 50 dB. A stable common-mode voltage is provided for each differential difference amplifier using an on-board voltage regulator, Analog Devices Inc. LT3021, and the voltage amount is equal to half of the ADC full-scale reference voltage ($\frac{A_{FS}}{2}=$0.6 V). Therefore, considering (6), adjusting the feedback matching ratio of the first amplification stage plays a critical role in the total readout CMRR.

The plots of the required percent component tolerance for various stage gain, $A_{v,dd}$, versus percent feedback ratio mismatch using (5), are shown in Figure 6b. Stages with higher allocated differential gain are less sensitive to component tolerances to obtain a given feedback matching ratio.

The peripheral surface-mount resistors and the capacitors of the differential amplification and filtering circuit were measured one by one in every purchased batch (containing at least 10 with 1% tolerance), and the ones with the closest values to obtain the matching ratio of better than 0.001% were mounted on the boards. The values of the resistors in the amplification and filtering stages to achieve a gain distribution of 30×10.6×10.6 are; $R_{F_{1}}=300\;k\Omega ,\;R_{I_{1}}=10\;k\Omega ,\;R_{F_{2}}=R_{F_{3}}=16\;k\Omega ,\;R_{I}{}_{2}=R_{I}{}_{3}=750\;\Omega ,{\;R}_{\mathrm{bb}}=R_{cc}=10\;k\Omega$.

#### 3.1.2. Noise Considerations and Filtering Unit

The input referred noise voltage, and current of the LTC6363 are 2.9 $\mathrm{nV}/\sqrt{\mathrm{Hz}}$ and 0.55 $\mathrm{pA}/\sqrt{\mathrm{Hz}}$, respectively. With the known gain distribution and resistance values of the amplification and filtering stages and typical values for the electrode impedance models, $R_{el}=200\;\Omega ,\;C_{el}=100\;\mathrm{nF}$ using (8) the output referred noise of the readout board before ADC is $75.231\;\mu V/\sqrt{\mathrm{Hz}}$. For an 8-bit resolution and 1% change in the impedance and $V_{AC}=50\;{\mathrm{mV}}_{\mathrm{rms}}$ the LSB of the differentially amplified sensing signal with the gain of 70 dB, is $3.34\;{\mathrm{mV}}_{\mathrm{rms}}$. Considering the fast response settling requirements, by picking an ENB of 270 Hz, the expected SNR at the minimum response level obtained from (9) is 7~9 dB. A bandwidth of 265 Hz is chosen for the bandpass filter, and with the available commercial component’s values for the capacitors $C_{b}=C_{c}=47.3\;\mathrm{nF}$, the center frequency of the filter is expected to be approximately 1.03 kHz. 

### 3.2. Real-Time Signal Acquisition and Sine Fitting

With the derived expressions in Section 2.2, the amplitude and phase estimation errors are good indicators of better noise immunity with various sample record lengths and specific accuracy requirements. To evaluate the reasonably achievable dynamic range for the sensor with the expected level of noise, jitter, and frequency accuracy, an optimized record length for a known M/N is required. Thus, theoretical derivations are verified with numerical MATLAB simulations. For the subsequent analysis, we assume two sine signals (source and response) with an equal frequency of 1 kHz, amplitude of 1 V, the relative phase difference of 45°, and equal DC offset of 0.6 V, that are generated using MATLAB. The sampling rate, $f_{s}$, and input frequency, $f_{in}$, are constant and equal to 55 kS/s and 1 kHz, respectively. The values M and N, are changed for multiple simulations, providing record lengths of 256, 512, and 1024 samples with 5, 9, and 19 cycles, respectively, to maintain the fixed M/N ratio. 

The ultimate metric of interest for a biosensor is to achieve a particular resolution. Therefore, the pointed non-idealities and their effect on 3PSF estimation are also examined here under the resolution (8-bit) context. The resolution of the sensor output is defined, considering a full-scale voltage at the input of the ADC, $A_{FS}$, and the full-scale target phase difference, $\varphi_{FS}$. One performance criterion given a target of 8-bit resolution is the estimation mean error and standard deviation remaining within the $\pm A_{FS}/2^{8}$ and $\pm \varphi_{FS}/2^{8}\;$range. The estimation errors (mean and standard deviation) and resolution lines are normalized to full-scale values to demonstrate a more generic reference plot. For each non-ideality effect, the normalized estimation parameters of interest with their percent mean error and percent standard deviation are shown, and normalized resolution lines are also drawn on the plots as an indicator of the best achievable resolution with different levels of additive noise, jitter, and shift in the excitation frequency.

#### 3.2.1. Additive Noise

**Figure 7:**
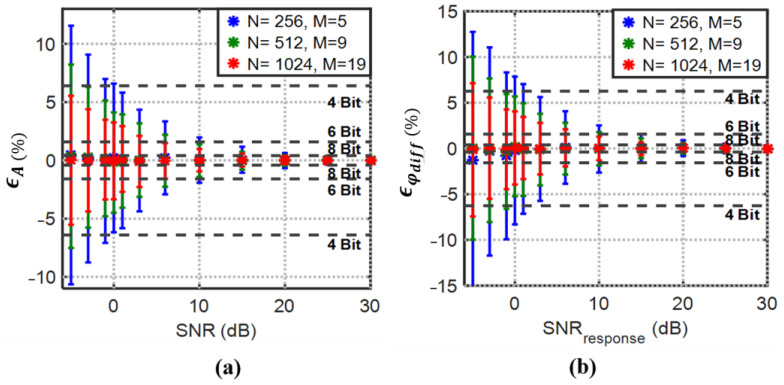
3PSF (**a**) percent mean amplitude and (**b**) percent mean differential phase estimation errors with their normalized standard deviations (error bars) vs. SNR. Normalized to full-scale resolution lines demonstrate the best achievable resolution by considering the standard deviation (error bars) with the degrading SNR and increasing record length (*N*).

The expected minimum SNR value at the output of the implemented amplification and filtering board is 7~9 dB. The analysis in Section 2.2.1 confirmed that an optimized record length for 3PSF improves both amplitude and differential phase estimation accuracy. To optimize the record length with the expected SNR, the estimation mean and standard deviation normalized percent errors obtained from the theoretical derivations (22)–(25) are simulated using MATLAB. Figure 7 shows the normalized mean amplitude and differential phase estimation error and the associated standard deviation for response SNR ranging from −5 to 30 dB and 1000 simulations at each point. The SNR at the source is fixed and set to 30 dB for the simulations. Apparently, with a larger record length, lower uncertainty in the estimation is achievable, even for SNR values less than 0 dB. 

The results in Figure 7 show that the 8-bit target resolution with SNR values even less than 5 dB is achievable with N=1024.

- Oversampling

Numerical analysis with varying sampling rates and low SNRs is also carried out for 3PSF to confirm if a higher sample rate leads to better estimation performance in a noisy environment. For the numerical simulations, the oversampling ratios range from 4 to 256, and the input SNR is assumed to range from −5 to 35 dB. The simulations for the 3PSF produce an average percent error within 1% both for the amplitude ratio and the differential phase for the 256 samples in the record. The produced estimation results show that with a lower sampling-to-excitation frequency ratio, 3PSF can deliver reliable results at very low SNRs, with even four samples taken per period. 

Although the greater number of samples within a record generates more accurate results with worse SNR levels, this might limit how fast the results could be produced at very low (sub-Hz) excitation frequencies. In [38], it is shown that utilizing 3PSF for a sub-Hz sensor response can produce an impedance estimation variance of 1% while the record covers only 11% of the whole period. Therefore, 3PSF is flexible for various excitation frequencies and signal-to-noise ratios. Consequently, it is concluded that at low SNRs, 3PSF with coherent sampling provides accurate results without oversampling. Therefore, the only limiting factor on the sampling rate is set by the real-time processing requirements.

#### 3.2.2. Jitter

**Figure 8:**
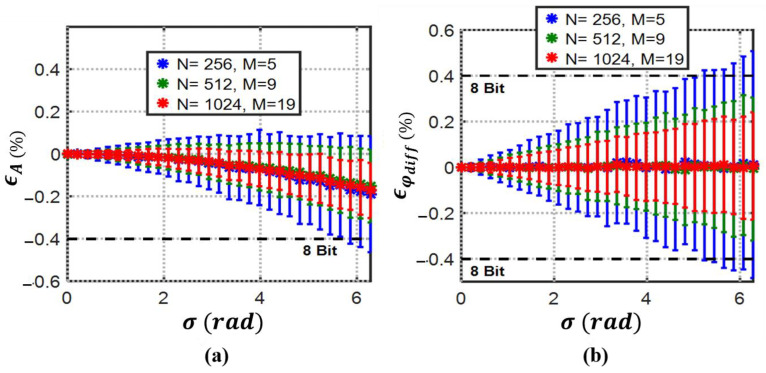
3PSF (**a**) percent mean amplitude and (**b**) percent mean differential phase estimation errors with their normalized standard deviations (error bars) vs. jitter standard deviation (σ). 3PFS can maintain 8-bit resolution with even unrealistic jitter standard deviations close to 2π by record length control at 55 kS/s sampling rate.

Based on derivations (31)–(34) for 3PSF in the presence of jitter and numerical simulations, the mean of the amplitude and differential phase errors with their normalized standard deviations for 1000 simulations at each point is plotted in Figure 8. The jitter standard deviation (σ) varies from 0 to 2π for all the simulations.

Although it is seen in (35) that the amplitude mean error will never reach zero for 3PSF [35], with a record length of 1024, the 8-bit detection resolution for both amplitude and phase is achievable even at non-realistically high jitter standard deviation of around π radians.

#### 3.2.3. Shift in Excitation Frequency

**Figure 9:**
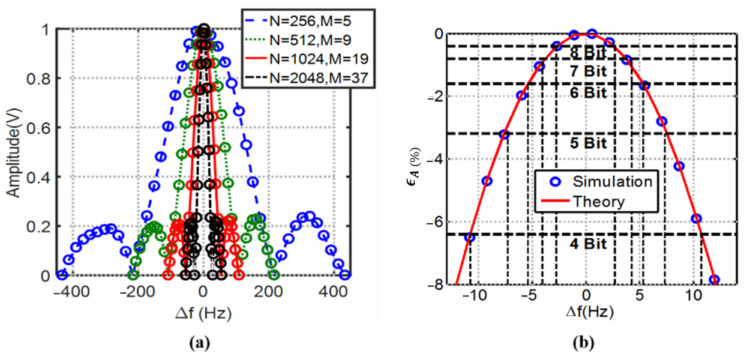
3PSF (**a**) amplitude estimation for a various number of samples within a record (**b**) zoomed-in Mean amplitude percent error for *N* = 1024, *M* = 19, and normalized percent detection resolutions vs. source frequency deviation. The plots also validate that the theoretical derivations for the non-coherency effect follow the numerical simulations accordingly.

Figure 9a shows the normalized amplitude estimation versus the deviation in the source frequency Δf at various numbers of acquired cycles (M=5, 9,19 and 37) both for the theoretical derivation (39) and numerical simulation, with $f_{s}=55\;\mathrm{kS}/s$ and $f_{in}=1\;\mathrm{kHz}$. For a higher numbers of samples, with approximately equal input frequencies and a constant sampling rate, the fitting accuracy becomes much more sensitive to uncertainty in the ratio of $f_{in}/f_{s}$. Figure 9b shows the zoomed-in plot of the amplitude error with non-coherency for N=1024, M=19. For 8-bit detection resolution, a frequency deviation of approximately 5 Hz can be tolerated, while if the resolution is relaxed to 4-bit, the safe frequency deviation is raised to 22 Hz. However, with the typical accuracy level of on-chip sine wave generators utilizing phase-locked loops (PLL) or direct digital synthesis (DDS) for discrete implementation, at kHz range, this amount of non-coherency is not a concern for amplitude estimation. 

However, at the MHz or GHz range, the accuracy of the generated sine wave is more of a limiting factor while picking the record length. 

**Figure 10:**
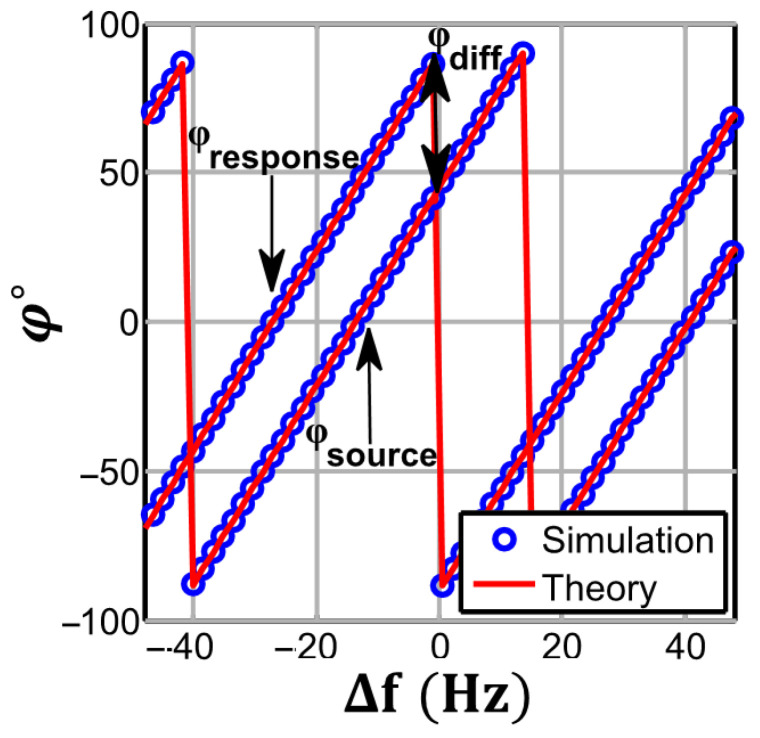
3PSF initial phase estimation vs. source frequency deviation for $\varphi_{source}=45{}^{\circ},$$\varphi_{response}=90{}^{\circ}$. The plots obtained for *N* = 1024, *M* = 19. When the frequency of the source and response signals is generated from a mutual source, the linear phase estimation error for both has the same slope.

The theoretical (41) and numerically simulated initial phase estimation of the source and response with non-coherency are shown in Figure 10. A deviation in the source frequency will result in a linear increase in the initial phase, but the slope of this change, as derived in (41), is equal for both source and response signals, as they share the same frequency from a mutual source. Therefore, the resultant differential phase estimation for the alternate sampling scheme will not be affected by frequency deviation for any record length.

It should be clarified that, as seen in Figure 9a, the estimated amplitude will reach zero when the shift in the frequency leads to an integer number of acquired cycles difference (i.e., δ=0 or when $∆f=\frac{Q}{N}f_{s}$, see (39)), causing a discontinuity in the initial phase estimation at the same points.

### 3.3. Board Characterization

The fabricated amplification and filtering board is characterized by measuring the total differential gain and common-mode-induced differential gain to verify the expected board total CMRR. For the differential gain measurement, the Audio Precision 2272 instrument is used to provide a very small differential sinusoidal input signal with a $0.1\;{\mathrm{mV}}_{\mathrm{rms}}$ amplitude. The magnitude of the differential voltage at the output of the filtering board is measured using a digital multimeter. The differential phase of the board output with reference to the source signal is measured by an oscilloscope. The gain magnitude and differential phase are measured and recorded for multiple frequency points at a range from 0 to 2500 Hz. Common-mode-induced differential gain is characterized with the same method, except for a $1\;V_{\mathrm{rms}}$ voltage applied to the inputs of the board. 

**Figure 11:**
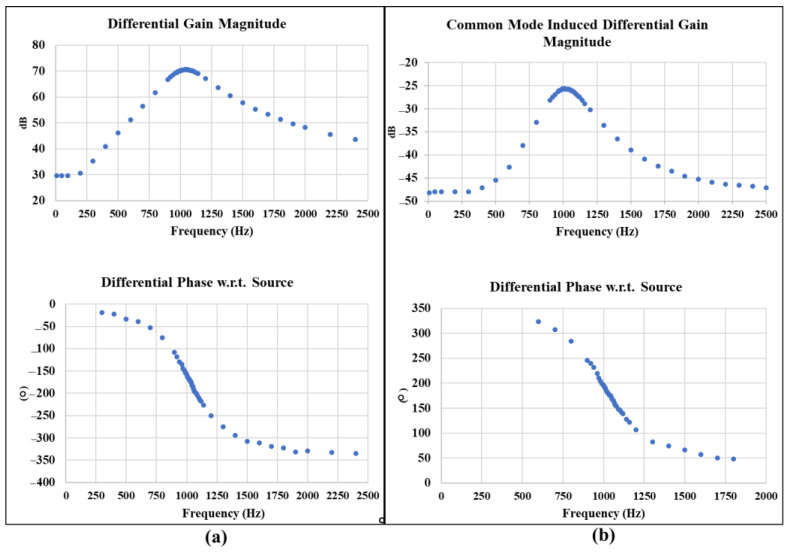
The amplification and filtering board characterization for differential gain magnitude and differential phase verifies a center frequency of 1.02 kHz, bandwidth of 265 Hz, and a differential gain magnitude of 3330.33 v/v  corresponding to 70.44 dB; (**a**). Maximum common mode induced differential gain magnitude is verified at −25.7 dB, which yields a total CMRR of 96.14 dB (**b**).

The measurement characterization graphs in Figure 11a show that the interface board can achieve a center frequency at 1.02 kHz, a maximum differential gain of 3330.33 v/v corresponding to 70.44 dB, and a bandwidth of 265 Hz. The maximum common mode induced differential gain shown in the measurement graph of Figure 11b is −25.7 dB, which yields a total CMRR of 96.14 dB.

### 3.4. Performance Verification

The amplification and filtering board is connected to the Texas Instruments Inc. MSP-EXP432P401R launchpad shown in Figure 5a for real-time data acquisition and sine fitting. The utilized key features of the single microcontroller are 48 MHz master clock rate, 14-bit, 1.2 V differential (two channel) ADC. Considering the acquisition of 1024 samples for each sine fitting operation satisfies 8-bit detection resolution requirements with 7~9 dB expected minimum SNR. The sampling rate is adjusted at 55 kS/s to accommodate the needed real-time processing time. For the real-time fitting operation, each sample is multiplied by the corresponding $\sin\left(2\pi f_{in}t_{k}\right)$ and $\cos\left(2\pi f_{in}t_{k}\right)$ from a lookup table and results consecutively added to produce the $\hat{A_{s}}\;$and $\hat{A_{c}}$ for each sine fitting.

#### Real-Time Operation and Sensitivity 

**Figure 12:**
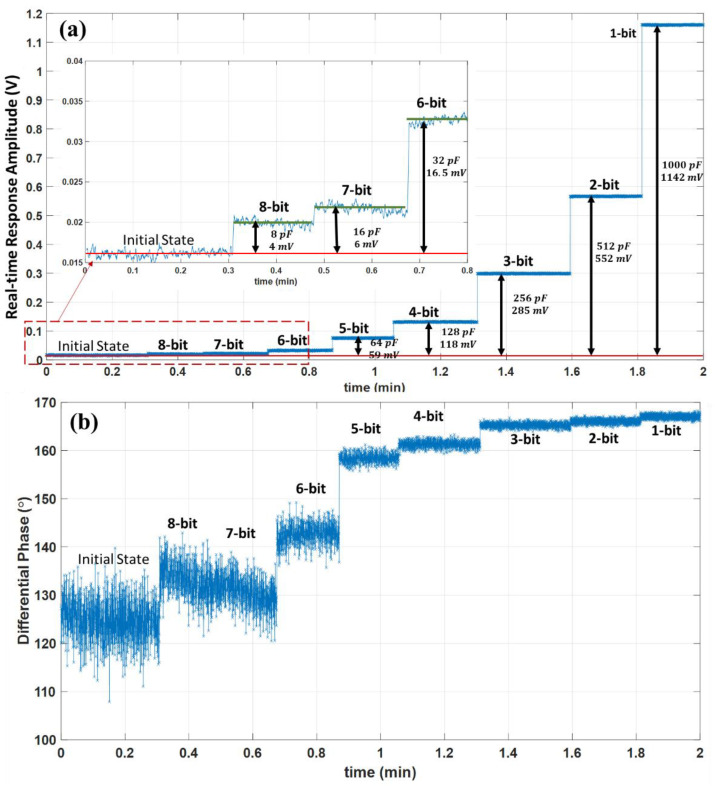
Sensitivity to 1% capacitance change with 8-bit resolution and real-time processing verification for the implementation of the readout board. The obtained amplitude from the real-time 3PSF for 8 pF, 16 pF, 32 pF, 64 pF, 128 pF, 256 pF, 512 pF and 1 nF capacitance change at a symmetrical impedance bridge with a fixed capacitance of 100 nF; (**a**). the obtained real-time differential phase and the corresponding amplitude can be used to compute actual capacitance change for a known bridge impedance model (**b**).

The readout sensitivity is tested using the same block diagram of Figure 5b. The test’s target is to detect a total 1% fractional capacitance change on one of the bridge impedances with an 8-bit resolution. A symmetrical bridge is configured by replacing $Z_{el1}$ and $Z_{el2}$ with the two fixed equal series RC impedances of 100 nF and 220 Ω. After balancing the differential bridge using digitally tunable $Z_{b1}$ and $Z_{b2}\;$networks, the 8-bit sensitivity is tested by additively changing the capacitance of the $Z_{b1}$ with 8 pF, 16 pF, 32 pF, 64 pF, 128 pF, 256 pF, 512 pF, and 1 nF values. The test mimics an 8-bit detection resolution for a total 1% ($\frac{1\mathrm{nF}}{100\;\mathrm{nF}}$) fractional change of the capacitance in the symmetrical differential bridge. The real-time response amplitude and differential phase with reference to the source signal are demonstrated in Figure 12.

The results show a distinct amplitude change at each consecutive capacitance change step compared to the initial balanced state amplitude. The change of amplitude values compared to the initial state at each binary weighted capacitance change is 4 mV, 6 mV, 16.5 mV, 59 mV, 118 mV, 285 mV, 552 mV, and 1142 mV, correspondingly from the 8-bit to a 1-bit resolution. The 8-bit, 7-bit, and 6-bit amplitude levels are shown in an inset zoomed-in plot of Figure 12a after a 10-point moving average. The real-time differential phase is shown in Figure 12b, together with the corresponding amplitude, can be used to algebraically compute the exact values of the capacitance change with the known bridge impedance model [20]. The obtained results in Figure 12 reveal that the readout is sensitive to a 1% capacitance change with an 8-bit resolution. Moreover, the results confirm that the developed interface can produce the processed response successfully in real time. The developed readout board could be interfaced with various custom-designed differential impedance sensing units with known impedance models, and the given design procedure could be employed for various precision-demanding applications.

## 4. Discussion

At the lower limits of detection for impedance biosensors, differential sensing and single frequency measurement with sufficient amplification and filtering are promising methods for real-time and field deployable implementation. However, when the response is still small and comparable to the noise level even after amplification, the resolution of the biosensor is significantly affected by the noise level. Moreover, the clock jitter will induce additional noise and degrade the SNR. Real-time digitization and processing with 3PSF, in expected low SNRs (even SNR < 0 dB), can lead to a higher resolution and better noise-immune operation. The algorithm can be implemented within a single MCU to process the digitized data in real time with an optimized number of samples per fitting to achieve a specific target resolution. With the assumption of a known operation frequency for the implementation of 3PSF, it is proven that a non-ideal shift in the source frequency and sampling clock jitter will affect the estimated response amplitude at any detection resolution. However, with typical accuracy levels of sine generation and the fact that both sampling clock and source signals are driven by a single sinusoidal source in most cases, jitter and source frequency shift will have a minor effect on the detection resolution both in terms of amplitude and differential phase at the lower sampling rates (i.e., tens to hundreds of KS/s). The real-time processing of the data obtained from the biosensor will eliminate the need for data storage and memory requirements and lead to lower costs for the overall system. A less complicated data processing algorithm with lower memory requirements, such as 3PSF, facilitates using the same microcontroller for data processing and other calibration or balancing of the differential system, e.g., the bridge-based system [20]. More straightforward data processing and less complicated readout implementation that are compatible to interface with ultra-sensitive transducers are crucial for the commercialization of cheaper biosensors.

## 5. Conclusions

A readout interface board suitable for high-precision impedance measurement, particularly for biosensing applications, is designed and implemented. The provided design details are first-hand knowledge for researchers in the field of impedance sensors and biosensors requiring precise measurement with specific resolution and accuracy. Moreover, the procedure provided here for developing a real-time data-acquisition unit is a guideline for making custom-designed, low-cost, and real-time digitization and processing units for numerous sensitive transducers that are currently being characterized with lab instrumentation. Therefore, utilizing the information provided in this paper for less complicated and yet accurate real-time readout facilitates the deployment of transducers for various in situ applications. 

## Data Availability

Not applicable.
